# Retraction Note: Breast cancer awareness among Sudanese females in Omdurman city: 2021

**DOI:** 10.1186/s12889-026-29482-8

**Published:** 2026-09-18

**Authors:** Munir Rafat, Mustafa Sabir

**Affiliations:** Faculty of medicine, AlNeelain University, Khartoum, Sudan

**Retraction Note: BMC Public Health 23, 1907 (2023)**

**https://doi.org/10.1186/s12889-023-16833-y**

The Editors have retracted this article because it it substantially overlaps with an article by the same authors that was published previously [1]. This article is therefore redundant.

Munir Rafat Munir Lawis did not state explicitely whether they agree or disagree with this retraction. Mustafa Sabir did not reply to any correspondence from Publisher regarding this retraction.
